# Inhibitors of Peptidyl Proline Isomerases As Antivirals in Hepatitis C and Other Viruses

**DOI:** 10.1371/journal.ppat.1004428

**Published:** 2014-11-06

**Authors:** Rob Striker, Andrew Mehle

**Affiliations:** 1 Department of Medicine, W. S. Middleton Memorial Veteran's Association, Madison, Wisconsin, United States of America; 2 Department of Medical Microbiology and Immunology, University of Wisconsin-Madison, Madison, Wisconsin, United States of America; 3 Department of Medicine, University of Wisconsin-Madison, Madison, Wisconsin, United States of America; University of Florida, United States of America

Viruses have small genomes with limited coding capacity. A common strategy by which viral genomes maximize their coding capacity is to express multifunctional proteins that promiscuously interact with various cellular partners to perform an array of essential functions. These interactions often involve flexible, and in some cases intrinsically disordered, viral domains or entire proteins that assume distinct conformations only upon binding cellular partners (see review, [1]). Viral coding capacity is further enhanced by relying on host factors and protein folding machinery to access different conformations and functions. These disordered peptide regions can be computationally recognized by features such as glycine, serine, and proline residues in contexts that are not conducive to β–strands or α–helices (reviewed by [2]). Bioinformatic analysis was used to predict the rigidity of proteins encoded by nearly 3,500 genomes from archaea, bacteria, eukaryotes, and viruses. This analysis suggests that almost all genomes with greater than 50% of their encoded residues in a predicted disordered state are viral genomes [3]. Thus, disordered proteins are enriched in the viral proteome and are common features to a large number of viruses.

Flexible viral proteins and/or domains interact with the cellular folding machinery, including proline isomerases. While proline is traditionally thought of as being a rigid amino acid that can “kink” the polypeptide chain, prolines can slowly rotate between two energetically similar configurations, *cis* or *trans*. This rotation is only fast enough to be physiologically relevant when facilitated by proline isomerases (rotamases) such as mammalian cyclophilins [4]. At least four structurally distinct classes of cellular proline isomerases exist in bacteria and eukaryotes, and some viruses encode their own proline isomerase [4], [5]. Identification of the host isomerases exploited by viruses and the viral proteins that require them to perform essential viral functions for replication in culture, or more importantly, in animals, presents an obvious antiviral strategy. Whether or not inhibition of host proline isomerases could be an antiviral strategy for hepatitis C virus (HCV) was a subject of debate for several years.

## What Is the Evidence That Cyclophilin A Is Needed in the HCV Life Cycle?

Cyclophilins are the most-studied proline isomerases and likely the least discriminating in terms of substrate choice. Cyclophilins were discovered as a target of the immunosuppressive drug cyclosporine. Cyclosporine, as well as immunosuppressant tacrolimus, both inhibit the adaptive immune response by inhibiting distinct classes of peptidyl proline isomerases; cyclosporine inhibits cyclophilins while tacrolimus inhibits FK506-binding proteins (FKBPs) [4]. A decade ago two groups showed that self-replicating HCV RNAs (replicons) are dependent upon HCV nonstructural proteins and are inhibited by cyclosporine, but not tacrolimus [6], [7]. This led to the proposal that HCV replication requires cyclophilins, but not FKBPs. This conflicted with observations by many clinical scientists. From 1983 until around 1998, when tacrolimus began supplanting cyclosporine, HCV-positive transplant patients received cyclosporine to prevent organ rejection, but this treatment did not simultaneously cure their HCV infection. Then and now, HCV is not only the most common reason for a liver transplant but also a common reason for needing a second liver transplant because immunosuppression (or the associated higher viral load that may arise from increased replication in the transplanted liver) clearly accelerated HCV-mediated disease in the liver graft. Thus, defining a role for proline isomerases during HCV infection in patients was confounded by the fact that any antiviral benefit from cyclosporine must overwhelm its immunosuppressive effect. Replicon data provided critical evidence for the role of cyclophilins during HCV infection in culture, yet cyclosporine and tacrolimus appeared to be “equally bad” for HCV-infected solid organ transplant patients; thus, controversy persisted [8].

There is now no longer a question that cyclophilin A plays a crucial role during HCV replication and cyclophilin inhibitors possess potent anti-HCV activity. Using an innovative mouse-human chimera model, it was shown that HCV replicates to significantly lower levels in cyclophilin A–deficient animals than in mice with cyclophilin A [9]. Several cyclophilin inhibitors that retain anti-HCV activity but do not have the immunosuppressive properties of cyclosporine and tacrolimus have also been studied. These compounds maintain their antiviral activity, and at least one has reached late Phase III trials for HCV [10]. Whether there is a role for cyclophilin inhibitors in future cures of HCV remains unclear. Multiple inhibitors that target viral enzymes and promote viral clearance in a high percentage of patients are being adopted [10]. Still, cyclophilin inhibitors may ultimately prove clinically useful for viral infections that resist new treatment regimes or useful when used in combination with existing therapies.

## On Which HCV Proteins Do Cyclophilins Act?

HCV encodes only ten proteins, including structural and nonstructural (NS) proteins. Cyclophilin inhibitors that reduce viral replication also block interactions between cyclophilin A and NS5A, suggesting that this association is important during the viral life cycle and might be the relevant target of the antiviral activity of cyclosporine [11]–[13]. An interaction between cyclophilin B and the viral polymerase NS5B was also reported [14]. Selection of subgenomic replicons for cyclosporine resistance created mutations in both NS5A and NS5B [15]. Mutating specific NS5B residues in isolation conferred low level or no resistance to cyclosporine [16], whereas mutations in NS5A, the most proline-rich HCV protein, conferred the highest levels of resistance [15]. These data suggest that NS5A is the most important substrate of cyclophilins for HCV replication. Additional work by multiple groups showed that cyclophilin A is the major, if not only, cyclophilin to play a role in the HCV life cycle [16], [17].

**Figure 1:**
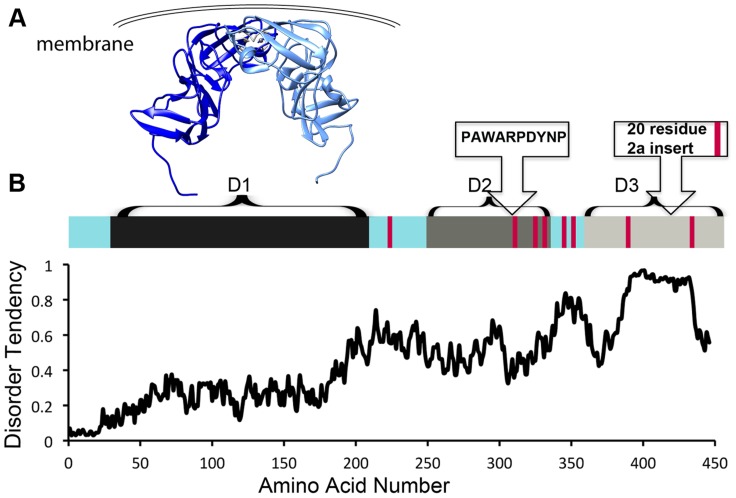
HCV NS5A is a protein that is rich in both proline residues and disorder and that associates with cyclophilin A via domain 2. A) Crystallographic model of domain 1 of NS5A (residues 37–213, PDB 1ZH1), which has a well-defined structure. Interestingly, a similar, but alternate structure for domain 1 with a completely different dimer interface (PDB 3FQM) has also been solved and it is currently unknown whether a single conformation or both best represent the intracellular state. B) Linear representation of NS5A. Current evidence suggests the entire carboxy terminus is disordered, but it has traditionally been studied as seperate domains termed domain 2 and 3 [11]. Red bars represent diprolines. Plot of NS5A disorder prediction from IUPRED (iupred.enzim.hu/).

NS5A is approximately 54 kD with three distinct domains separated by low-complexity sequences (Figure 1). Approximately 10% of the amino acids in NS5A are prolines. In addition, NS5A has regions of high disorder, multiple reversible posttranslational modifications, and helical tendencies [18]. NS5A displays significant sequence variability across viral genotypes, yet it typically contains approximately 8 Pro-Pro motifs. It is not clear if these conserved diprolines are biologically relevant cyclophilin A-interacting sites. Mapping studies have implicated the residues in domain 2 as critical for the effects of cyclophilin A and cyclophilin inhibitors during viral replication (Figure 1A, 1B) [11]–[13]. Nuclear Magnetic Resonance (NMR) studies indicate that much of domain 2 is disordered [18]. Additionally, NMR studies show that several prolines in domain 3 occupy both *cis* and *trans* configurations and may thus be substrates for isomerases [18]. Domain 3 from genotype 2 strains also contains a proline-rich insert (Figure 1B). But, despite the presence of the proline-rich insert at least one genotype 2 strain has less cyclophilin dependence [19], suggesting that specific proline context, rather than the number or percentage of proline residues may determine the importance of cyclophilin and whether cyclophilin inhibitors are an applicable antiviral strategy.

Within NS5A domain 2, most evidence implicates a single proline (P319) and the tryptophan, aspartate, and tyrosine residues surrounding it in a WARPDYN motif as being especially significant [11], [13], [20]. The WARPDYN motif itself is bracketed by additional proline residues (P[A/I]WARPDYNP). Mutations conferring resistance to cyclophilin inhibitors map to the WARPDYN motif, e.g., R318W and D320E [13]. The D320E mutation had little to no effect on the binding of NS5A to cyclophilin A. However, even though this is a conservative change, the D320E mutation appears to alter the local protein conformation. NMR spectra of a 20-amino-acid peptide that includes the prolines bracketing the WARPDYN motif showed that the isomerization state of P319 exists in equilibrium with approximately 75% in the *trans* conformations. Conversely, spectra collected on peptide containing the resistance mutation D320E revealed that approximately 70% of P319 was now in the *cis* conformation. Thus, mutations that confer resistance to cyclophilin inhibitors shift the *cis∶trans* ratio of configurations in the motif, reducing dependence on the isomerase activity of cyclophilins [13]. Cyclophilin A has at least low-level affinity for multiple other stretches of domain 2, including two tripeptide alanine-hydrophobic residue-proline motifs [20] that surround the WARPDYN. Additional mutations adjacent to the WARPDYN motif arise in patients treated with cyclosporine. An atypical proline (P328, which is the consensus amino acid in only 5% of genotype 1 strains) downstream of the motif was detected in one patient prior to treatment that mutated to serine following exposure to cyclosporine [21]. The NS5A P328 variant possessed enhanced susceptibility to cyclosporine in replicon experiments that was lost upon mutation to serine, suggesting in at least this patient a concentration of cyclosporine was achieved in vivo that had an antiviral effect [21]. These data identify critical regions in NS5A that recruit and utilize cyclophilin A to participate in viral genome replication.

## Do Disordered Regions of a Proline-Rich Viral Target Contribute to the Viral Protein Being a Substrate for Cyclophilins?

**Figure 2:**
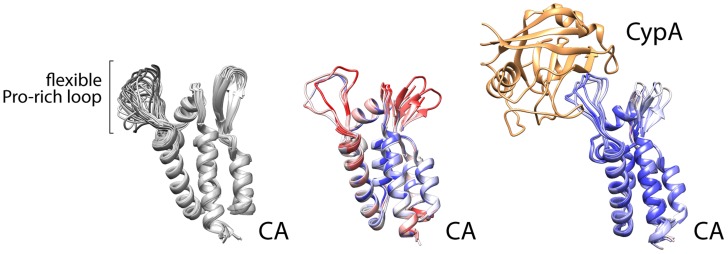
Cyclophilin A binds a flexible loop in the HIV CA, containing glycine 89 and proline 90 and flanked by prolines. NMR solution structures (left, 1GWP) and multiple crystallographic models of CA (center, 1MG3, 1E6J, 3P05) highlight the structural variability of the Pro loop that is minimized by Cyclophilin A binding (right, 1AK4, 1M9C, 1M9D). Crystal structures are colored by B-factor (a measure of thermal motion and disorder within the structure) as a proxy of conformational flexibilty (red = disordered, blue = ordered). NB: the Pro loop is resolved in only three of the seven protomers in the crystal structure in the unbound state.

Despite the depth of information regarding NS5A domain 2, our basic understanding of which viral prolines in HCV or other viruses require isomerases is limited. This is in part because only one other example has been investigated extensively—the association between cyclophilin A and capsid (CA) from the HIV Gag protein [22]. That interaction was captured in crystal structures, revealing that cyclophilin A binds a relatively flexible loop between structured parts of the viral CA (Figure 2) [23]. It is certainly premature to draw general conclusions about viral–cyclophilin interactions when only two have been characterized to any depth, but some noteworthy similarities and differences can be made. The HIV CA loop contains a single glycine-proline motif, with the proline (P90) existing in both *trans* and *cis* conformations in different structures and in solution [22], [24]. The lack of structural rigidity for this loop is exemplified by the multiple conformations detected in solution structures, the higher B-factor for this region of the protein in crystal structure without cyclophilin, or disorder and the lack of structural information in some models (Figure 2). The glycine-proline motif of CA has no obvious resemblance to the cyclophilin A interaction site in HCV NS5A. However, these regions share several common properties: the flexible cyclophilin-interacting loop in CA is also bracketed by prolines, just as the PAWARPDYNP motif in HCV; residues 86, 91, and 96 that surround the glycine-proline in CA influence viral susceptibility to cyclophillin inhibitors similar to mutations surrounding NS5A P319 [25]; and the critical glycine-proline is amino-terminal to regions of CA that are actually more proline rich and predicted by bioinformatics analysis to be disordered, analogous to the positioning of the PAWARPDYNP motif in HCV NS5A. The interaction of HIV CA with cyclophilin A leads to packaging of cyclophilin A into the viral particle. Yet, cyclophilin A appears to function primarily in capsid uncoating [25], rather than particle assembly, and does not have a role in HIV genome replication as it does for HCV. While limited, this comparison provides common themes that may facilitate the identification of other viral proteins that rely on host proline isomerases for function and may thus be susceptible to intervention by blocking isomerization. The HIV Gag protein also contains proline stretches termed proline-rich motifs (PRMs) [26], but they do not appear to be critical targets for cyclophilin A. While some proline content favors disorder, consecutive prolines impart rigidity. PRMs generally have proline content surpassing 30%, and neither viral nor human PRMs have yet been described interacting with cyclophilins specifically.

New viral threats emerge much faster than rationally designed antivirals. The number of clinically useful antivirals remains limited, so a drug that works on multiple viruses would be welcomed. Identifying viruses and viral proteins that depend on host proline isomerases is an appealing strategy. For example, at least some lethal coronaviruses are suppressed by cyclophilin inhibitors [27]. Unfortunately, merely identifying a proline-rich viral protein is not sufficient to predict interactions with isomerase or sensitivity to isomerase inhibitors. Creative, systematic approaches are needed to determine which viral proteins contain prolines that are substrates for isomerases and access multiple conformations to perform critical viral functions.
